# Poor Treatment Response in Panic Disorder Patients with Suicide Attempts and Their Symptom Network Characteristics

**DOI:** 10.1155/2023/5194900

**Published:** 2023-05-04

**Authors:** Hyun-Ju Kim, Hye-Yeon Jung, Minji Bang, Chongwon Pae, Sang-Hyuk Lee

**Affiliations:** Department of Psychiatry, CHA Bundang Medical Center, CHA University School of Medicine, Seongnam, Republic of Korea

## Abstract

**Background:**

Panic disorder (PD) is associated with suicidality. Depression has been suggested as a link between PD and suicide; however, this remains controversial. Comprehensive research on the history of suicide attempt (SA) in patients with PD is scarce. We investigated the clinical characteristics of SA in patients with PD using PD-related assessments and network approaches.

**Methods:**

A total of 1151 participants were enrolled, including 755 patients with PD (97 with SA (PD+SA) and 658 without SA (PD-SA)) and 396 healthy controls. The Scale for Suicide Ideation and Panic Disorder Severity, Anxiety Sensitivity Inventory, and other PD-related measures were also administered. We compared symptom severity and analyzed the pharmacological treatment response in patients with PD with and without SA. Network analysis was used to estimate the centrality, stability, and network structures of the nodes.

**Results:**

Our results revealed that the scores for panic and depressive symptoms, pathological worry, anxiety sensitivity, and the frequency of early trauma were significantly higher in the PD+SA group than in the PD-SA group. Multiple linear regression analysis revealed that short- and long-term pharmacological treatment responses were significantly poorer in the PD+SA group. Network analysis showed that fear of cognitive dyscontrol (FCD), as a cognitive aspect of anxiety sensitivity, was the central symptom through strength, expected influence (one and two steps), randomized shortest path betweenness, and eigenvector centrality measures in the PD+SA group. In contrast, depression was the central symptom of patients with PD-SA.

**Conclusion:**

Our study suggests that a history of SA could be associated with high panic-symptom severity and poor pharmacological treatment response in patients with PD and that FCD is the most central symptom in the PD+SA network. Central symptoms, such as cognitive aspects of AS in patients with PD+SA, may be clinically effective as potential targets for intervention in patients with PD at risk of or suffering from suicidality.

## 1. Introduction

Suicide has become a critical public health concern worldwide. The rate of suicide in South Korea is among the highest in the Organization for Economic Co-operation and Development [1]. A suicide attempt (SA) can be defined as a behavior of self-injury with the intention to die, and it is also a strong indicator of completed suicide [2, 3]. Thus, SAs may be an area for clinical intervention and a marker for preventing future suicide because they might be a marker of future reattempt or suicide [4].

Panic disorder (PD) is characterized by recurrent panic attacks, anticipatory anxiety, agoraphobia, and related functional impairments [5, 6]. A study showed that patients with PD are 4.39 times more likely to have suicidal ideation and 3.96 times as likely to commit suicide than individuals without PD [7].

Some clinical variables, including early-onset age, female sex, and comorbidities (such as generalized anxiety disorder and mood disorders), may play a partial role in the increase in suicide risk in patients with PD [8–10]. In addition, pathological worry-related anxiety as measured by the Penn State Worry Questionnaire (PSWQ) was significantly correlated with suicide risk [11, 12].

Among various clinical factors, it has been proposed that the coexistence of depressive disorders may explain increased suicidality in patients with PD for a long time [13, 14]. However, other researchers have reported that the risk of suicidality in patients with PD cannot be fully explained by the comorbidity of depressive disorders [15]. Goodwin and Roy-Byrne showed that postyear SAs were independently associated with prior 12-month and lifetime PD even after controlling for the effects of the comorbid depression [16]. Compared with those with depressive disorders, patients with PD also have high mortality rates similar to those of patients with depression. It can be inferred that they experience death by complete suicide or cardiovascular disease, though they are more likely to seek help from clinicians [17, 18].

Another suggested vulnerability factor for increased suicide risk is anxiety sensitivity (AS) in patients with PD [19]. AS is simply referred to as a “fear of anxiety sensations” and consists of cognitive, social, and physical components, such as cardiovascular or respiratory symptoms [20]. Clark's cognitive model showed that patients with PD tended to have a cognitive bias when interpreting certain bodily sensations related to threats or dangers [21, 22]. A previous study has insisted that there were cognitive aspects of AS, or fear of cognitive dyscontrol (FCD), that influences the links between PD and SA, with the largest effect size compared to the social or physical AS subscales [19]. FCD is a cognitive aspect of AS and is defined as the fear of dying during a panic attack (e.g., trembling) [23]. Interestingly, it seems that patients experiencing FCD amplified the distress responses triggered by unpleasant feelings, which facilitate SA when this amplification reaches a severe stage [24]. Moreover, some researchers often insisted that the FCD in PD may be an independent risk factor for SAs even after comorbid psychiatric disorders and demographic characteristics are considered together [19, 25]. Therefore, further research is needed to investigate the relationship between depression or AS (especially, cognitive AS) and suicidality in patients with PD.

In general, a history of a SA is regarded as the most important and robust predictor of completed suicide [3, 26]. Moreover, a previous study on PD with SA showed that SA history is associated with a high level of AS, especially with social concerns, poor panic outcome-physical, high social functional impairment, and high overall disability [13]. These findings suggest that SA history might strongly predict future completed suicide and poor prognosis, resulting in high social impairment and overall disability in patients with PD. Furthermore, a previous study on patients with depression suggested that SA history can be associated with an earlier age of onset, longer duration of illness, more severe psychopathology, and poorer treatment outcomes [27]. However, no study has been conducted on the association between treatment outcomes and the history of SA in PD.

Meanwhile, several network-based studies have received considerable attention. A network approach to psychopathology is a set of integrated techniques that reveals the relationships and essential factors among diverse symptoms [28]. Centrality, which indicates the importance of the symptom, may have a more significant effect on the system [29, 30], and central symptoms have been suggested to contribute to the activation of correlated symptoms within the network. Additionally, the central nodes in a symptom network could be a potential starting point for therapeutic interventions [31]. Therefore, investigating the effects of SAs on PD in terms of symptom networks could support the identification of central symptoms and factors related to SAs. This may also provide a new perspective on the diagnosis and treatment effectiveness of PD. Since medication use can influence the symptom severity and the centrality indices of patients in the network analysis [32, 33], we need to evaluate the patients after excluding the medication effects.

This study investigated the characteristics and pharmacological treatment responses of patients with PD with or without SAs. Moreover, a network analysis was conducted to investigate the central symptoms and their connection to suicidality among SA-related variables with and without SAs in unmedicated patients with PD. We hypothesized that (1) there would be an early-onset age and more severe PD-related psychopathology in the PD+SA group than in the PD-SA group and (2) lower pharmacological treatment responses with longitudinal follow-up in the PD+SA group than in the PD-SA group. (3) In the unmedicated PD symptomatology and suicidality network, FCD and depression markedly influenced the PD+SA network.

## 2. Methods

### 2.1. Participants

**Figure 1:**
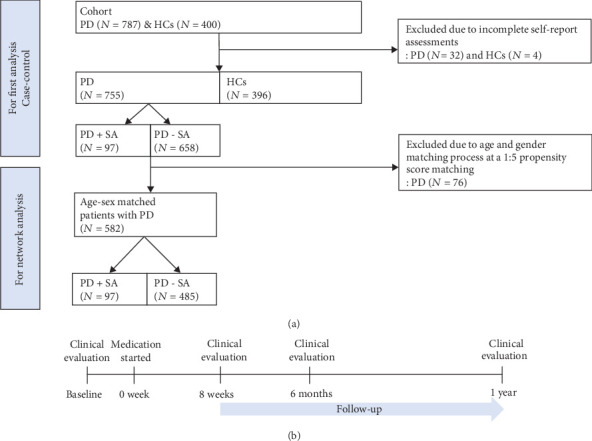
Flowchart (a) and timeline of the study (b). Abbreviations: PD: panic disorder; HCs: healthy controls; SA: suicide attempt.

The participant selection process is illustrated in Figure 1(a). The initial data included 1187 participants (787 patients diagnosed with PD and 400 healthy controls (HCs)). Patients with PD were followed up for up to 1 year after the commencement of pharmacotherapy. Thirty-two patients with PD and four HCs were excluded due to incomplete self-report assessments, and 755 patients with PD (360 men and 395 women, aged 17–70 years, mean = 38.78 ± 11.40 years) and 396 HCs (187 men and 209 women, aged 16–70 years, mean = 37.60 ± 10.87 years) remained. Additionally, 76 patients with PD were excluded by age and sex matching at a 1 : 5 ratio with propensity score matching in the network analysis because of the large difference in proportions between PD+SA and PD-SA groups. Ultimately, 582 patients with PD (248 men and 334 women, aged 16–65 years, mean = 35.74 ± 10.41 years) were included in the network analysis.

Patients with PD were recruited from the Department of Psychiatry of the CHA Bundang Medical Center (Seongnam, Republic of Korea) between December 2013 and June 2022. The HCs were registered in the local community using online and print advertising. Through individual interviews, trained psychiatrists confirmed that the HCs had no personal history of psychiatric disorders.

Patients with PD satisfied the criteria described in the Diagnostic and Statistical Manual of Mental Disorders, Fifth Edition (DSM-5) or Fifth Edition-Text Revision (DSM-5-TR), based on diagnoses proposed by trained psychiatrists using the Structured Clinical Interview based on the DSM-5 (SCID-5) [5]. We only included patients with PD with or without agoraphobia in the principal diagnosis, even though they had depressive disorders as an additional diagnosis, since the PD with/without agoraphobia mainly cooccurs with major depression in the natural course of PD and the aim of our study was to investigate the key clinical presentations of PD except other anxiety disorders. The exclusion criteria were as follows: (1) other major psychiatric comorbidities, including anxiety disorders other than PD, schizophrenia spectrum and other psychotic disorders, major depressive disorder with psychotic features, bipolar and related disorders, and substance-related and addictive disorders; (2) neurodevelopmental disorders; (3) neurocognitive disorders; (4) major medical disorders; and (5) pregnancy. Additionally, all patients with PD treated with individual or group psychotherapy, including mindfulness-based cognitive therapy or cognitive behavioral therapy (CBT), were excluded from the study.

Figure 1(b) shows the timeline for the evaluation of PD patients. We measured the symptom severity of patients with PD at their baseline unmedicated status. After baseline, all patients with PD received pharmacotherapy with antidepressants, including paroxetine, escitalopram, or sertraline (escitalopram equivalence dosage = 10.23 ± 7.06 (mean ± SD) mg/day) [34], and benzodiazepine (BDZ) as alprazolam or clonazepam was primarily permitted on a pro re nata (as required) basis. Some patients with PD undergo pharmacological treatment with antidepressants and anxiolytics according to the Korean medication algorithm for PD [35] or the Clinical Practice Guidelines: Treatment of PD [36]. Clinical interviews and several instruments were conducted during each patient's first visit to the hospital to evaluate the various factors affecting pharmacological treatment response in PD.

These study protocols were reviewed and achieved approval by the Institutional Review Board of CHA Bundang Medical Center. All study procedures were done according to the latest version of the Declaration of Helsinki and the principles of Good Clinical Practice. All participants submitted written informed consent.

### 2.2. Assessments

#### 2.2.1. Suicidality

A history of SA was defined as a self-reported history of one or more self-destructive behaviors with some degree of intent to end one's life before the baseline assessment [2]. Trained psychiatrists performed semistructured clinical interviews to confirm whether the patient had a presence of SA regarding the medical severity of attempts and suicidal intentions. The main interview question was “Have you ever committed suicide for any purpose in your lifetime?”. Furthermore, a self-reported assessment of the Scale for Suicide Ideation (SSI) [37] was used to measure the intensity of each participant's suicidal ideation, such as specific attitudes, behaviors, and plans to commit suicide. The SSI contains 19 items, each with three parts rated on a three-point scale from 0 to 2 according to the severity of suicidal thoughts and related symptoms (total scores: 0 to 38). The higher the total scores, the higher the risk of suicide, reflecting the need for clinicians to pay attention to these patients. The Korean version of the SSI has adequate internal consistency (Cronbach's alpha = 0.88) [38].

Trained psychiatrists evaluated participants' suicide risk using semistructured clinical interviews and self-reported assessments and classified them according to severity (e.g., low/moderate/severe). Clinicians made decisions according to the severity of the evaluated suicide risk. If participants were at or above moderate risk, clinicians encouraged them to seek social support, provided emergency contacts, and frequently contacted and educated them to come to the hospital immediately in case of an emergency [39]. It was also explained that if the participant had a severe suicide risk, voluntary and involuntary hospitalization should be considered.

#### 2.2.2. Symptomatology

All participants were rated for the clinical symptom intensity of PD by the self-report version of the Panic Disorder Severity Scale (PDSS) [40]. The PDSS consists of 7 items coded on a 5-point scale (0–4). The total scores ranged from 0 to 28. The internal consistency of the Korean version of the PDSS is good (Cronbach's alpha = 0.88) [41]. To assess the severity of depressive symptoms, we used the Beck Depression Inventory-II (BDI-II), which has high internal consistency (Cronbach's alpha = 0.91) [42, 43]. For supplementary evaluation of suicidality, we used the BDI-II item 9 (suicide item), which assesses the severity of suicidal thoughts [44]. This suicide item in the BDI exhibited a significant correlation with the SSI total scores in our study (*r* = 0.78, *p* < 0.001).

To assess potential trait markers in patients with PD, we used the Korean version of the Anxiety Sensitivity Inventory-Revised (ASI-R) [45], which consists of (1) fear of respiratory symptoms, (2) publicly observable anxiety reactions, (3) cardiovascular symptoms, and (4) cognitive dyscontrol. The internal consistency of the Korean version of the ASI-R is 0.93 [46].

To measure pathological worry, we administered the Korean version of the PSWQ, which showed high internal consistency (Cronbach's alpha = 0.95) [47]. Early trauma was assessed using the Korean version of the Early Trauma Inventory Self Report-Short Form (ETISR-SF) [48] at baseline. Cronbach's alpha of the Korean version of the ETISR-SF was 0.87.

The treatment response of patients with PD after pharmacotherapy was evaluated through the follow-up assessments of the PDSS at 8 weeks, 6 months, and one year from the pretreatment baseline. We defined the treatment response as the percentage of reduction at each period in the total PDSS score after 8 weeks, 6 months, and one year compared to the pretreatment baseline total PDSS score.

### 2.3. Statistical Analysis

Baseline demographic variables were compared between patients with PD and HCs and between PD patients with and without SA using independent *t*-tests and chi-square tests. The total scores of the PDSS, BDI-II, PSWQ, ASI-R, and ETISR-SF were compared using an analysis of covariance (ANCOVA) to control for age. In addition, multiple linear regression analysis with pharmacological treatment response as the dependent variable was conducted, controlling for confounding variables such as sex, age, and significant clinical assessment variables. Statistical analysis was performed using the Statistical Package for the Social Sciences version 26.0 (IBM Corporation, Armonk, NY, USA) to verify the results. In all analyses, a *p* value lower than 0.05 was considered significant.

### 2.4. Network Analysis

Among patients with PD, network analysis was conducted regarding network estimation, network accuracy and network stability, and comparison of networks between PD patients with and without SAs using the R software.

#### 2.4.1. Network Estimation

Network models were estimated separately using R software to analyze the relationship between nodes among patients with PD with and without SAs [49]. The network was estimated using the graphical least absolute shrinkage and selection (gLASSO) model with an extended Bayesian information criterion [50, 51] to determine the association between PD-related symptoms.

It has been suggested that the hypothesis of clinicians or psychiatrists based on empirical approaches is crucial when clinicians choose a set of nodes in a network analysis [52]. According to our hypothesis, in particular, our study is aimed at investigating how AS could be associated with SAs in patients with PD considering the network of panic-related symptoms (levels of depression, panic symptoms, and worry).

In this study, we sought to identify the overall association between suicidality based on a detailed perspective of AS. Our study included eight nodes for network estimation within the patients with PD group: one panic node (total scores of the PDSS), four AS node (scores of the ASI-R-fear of respiratory symptoms, publicly observable anxiety reaction, cardiovascular symptoms, and cognitive dyscontrol, i.e., FCD) subscales, one depressive node (total scores of the BDI-II), one pathological worry node (total scores of the PSWQ), and one suicidality node (BDI-II item 9).

In each network, four centrality indices, such as (1) strength, (2) expected influence (EI), (3) randomized shortest paths betweenness centrality (RSPBC), and (4) eigenvector, were used to determine nodes that were central and performed essential roles [53–55]. Node strength is defined as the absolute sum of the edge weights from a target node to the adjacent nodes. EI is the sum of all the edge weights emanating from a specific node, including negative connectivity. Both one-step and two-step effects can be computed using EI. As implied by its name, the two-step EI evaluates the connection between a node and up to two edges. Betweenness denotes the number of shortest pathways between two other nodes. A node with higher betweenness can be described as being more frequent in the paths that link one node to another. RSPBC is betweenness centrality based on the randomized shortest paths of each node in a network. The eigenvector, which measures the degree of connection to other central nodes, indicates the relative scores of all nodes in the network and influences the power of the network's hub.

#### 2.4.2. Network Stability and Accuracy

The bootstrap method was performed to determine the network robustness (centrality stability and edge weight accuracy). The accuracy of the edge weights was calculated using 95% confidence intervals (CIs), along with bootstrapped mean edge weights [50, 56] based on 10,000 bootstrap samples. The correlation stability coefficient (CS-C), an index representing the stability and reliability of centrality indices, was calculated to measure the stability of the selected centrality measurements using subset bootstrapping [57]. A CS-C of 0.5 or higher is a reliable measurement and should be at least 0.25 [50, 58]. Bootstrapped edge weight difference tests were used to evaluate the contrast between the edges [50].

#### 2.4.3. Comparison of Networks between PD Patients with and without SAs

To analyze network differences between PD+SA and PD-SA, we conducted a network comparison test using the R package *NetworkComparisonTest* [59]. To demonstrate the differences in invariance measures, including edge weights, strength, and EI, a network comparison was performed with 10,000 random permutations [60, 61]. Internetwork comparison for eigenvectors and RSPBC was also performed according to the method suggested by the *NetworkToolbox*. Furthermore, significant differences between the two networks were analyzed separately using a *p* value < 0.05 (two-tailed) significance level. To deal with the multiple comparison problems, a false discovery rate (FDR) correction was done (*q* < 0.05).

## 3. Results

### 3.1. Comparisons of Sociodemographic and Clinical Characteristics between the Patients with PD and HCs

The sociodemographic and clinical characteristics of the participants are summarized in Table 1. In the HCs, there were more people living without partner than patients with PD. Patients with PD showed significantly higher suicidality (SSI), panic symptom severity (PDSS and PSWQ), levels of depression (BDI-II), AS (ASI-R), and frequency of early trauma (ETISR-SF) than HCs after controlling for marital status.

### 3.2. Comparisons of Sociodemographic and Clinical Characteristics between the Patients with and without the History of Suicide Attempt

Table 2 presents the sociodemographic and clinical characteristics and age at the onset of PD+SA and PD-SA. There were no significant differences in the demographic characteristics between the PD+SA and PD-SA groups, except for age. However, the PD+SA group showed a significantly earlier age of onset of PD symptoms, high frequency of agoraphobia, and suicidality scores, including SSI total scores and a suicide item (BDI-II item 9), than the PD-SA group. In the ANCOVA controlling for age, significant differences were maintained.

### 3.3. ANCOVA Analyses for Comparison of Symptom Severities between Patients with Panic Disorder with and without Suicide Attempts

Table 3 shows the comparisons of symptom severity between the PD+SA and PD-SA groups using ANCOVA analyses. Additionally, the baseline total PDSS and BDI-II scores were significantly higher in the PD+SA group than in the PD-SA group (*F* = 20.06, *p* < 0.001 and *F* = 89.20, *p* < 0.001, respectively). The ANCOVA controlling for age demonstrated statistically significant differences in the PSWQ (*F* = 9.67, *p* < 0.001), ASI-R (*F* = 34.55, *p* < 0.001), and ETISR-SF (*F* = 27.01, *p* < 0.001).

### 3.4. Multiple Linear Regression Results Predicting Pharmacological Treatment Response in Patients with Panic Disorder

Multiple linear regression analyses controlling for confounding variables such as age, sex, and total scores of the PDSS, BDI-II, PSWQ, ASI-R, and ETISR-SF were performed to predict pharmacological treatment response in PD.  Table 4 summarizes the results of the three models. The research model for pharmacological treatment response at eight weeks was significant (*F* = 5.40, *p* < 0.001). The research models for pharmacological treatment response at 6 months and one year also showed significance (*F* = 4.47, *p* < 0.001 and *F* = 4.41, *p* < 0.001, respectively). The explanatory powers of these models were 19%, 20%, and 22% (*R*^2^ = 0.19, *R*^2^ = 0.20, and *R*^2^ = 0.22, respectively). There were significant negative associations between SAs and pharmacological treatment response at 8 weeks (*β* = −0.19, *p* = 0.011), 6 months (*β* = −0.20, *p* = 0.015), and one year (*β* = −0.28, *p* = 0.002) in patients with PD after controlling for confounding variables.

### 3.5. Network Analysis and Group Comparison

#### 3.5.1. Network Structures of PD Patients with and without Suicide Attempts

Table 5 indicates the PD+SA and PD-SA group demographics and the frequency of their responses to the PDSS, BDI-II, PSWQ, and ASI-R.

**Figure 2:**
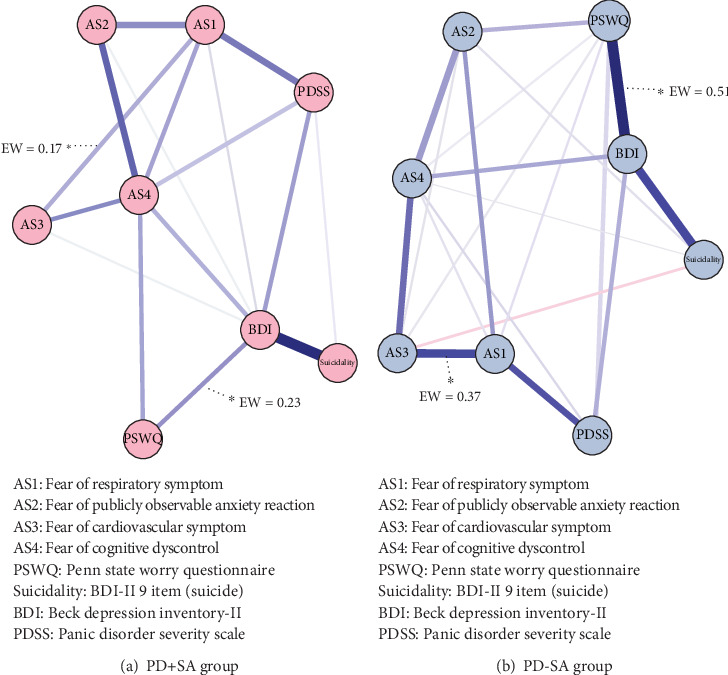
Network structures (a) with and (b) without the history of suicide attempts in patients with panic disorder. Note: the blue edges indicate positive correlations, and the red edges represent negative associations between two nodes. Asterisks (⁣^∗^) indicate the edge weights, which show significant differences between the two networks. The thickness of the edges was proportional to the strength of the correlation. Abbreviations: PD: panic disorder; SA: suicide attempt; EW: edge weight.

The PD+SA network yielded 28 edges, of which 16 edges had nonzero weights, whereas the PD-SA network showed nonzero 21 edges out of 28 edges (Figure 2). In the PD+SA network, the strongest edge between nodes was the connection between suicidality and BDI (*r* = 0.23), and the second strongest edge was the connection between AS2 (fear of publicly observable anxiety reaction) and AS4 (FCD) (*r* = 0.17). In contrast, in the network of PD patients without SA, BDI–PSWQ (*r* = 0.51) was followed by suicidality and BDI (*r* = 0.38).

**Figure 3:**
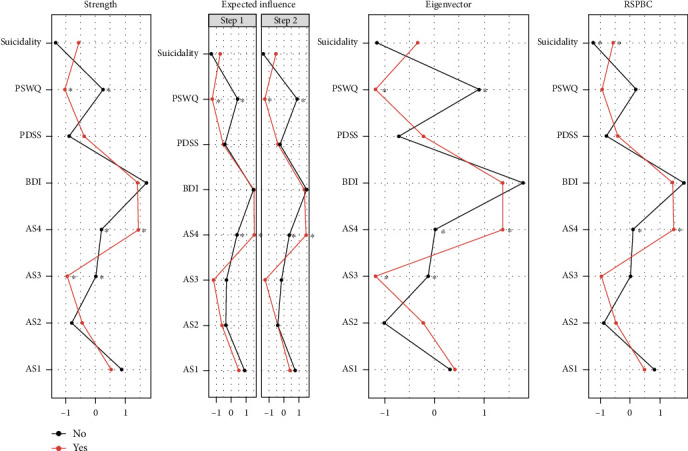
Centrality index (strength, expected influence, eigenvector, and RSPBC) plots in patients with panic disorder with and without a history of suicide attempts. Note: all plotted values are standardized *z*-scores. Nodes with significantly different centrality measures between the two networks in patients with PD are shown by asterisks (⁣^∗^). The “yes” and “no” mean the history of a suicide attempt. Abbreviations: AS1: fear of respiratory symptom; AS2: fear of publicly observable anxiety reaction; AS3: fear of cardiovascular symptom; AS4: fear of cognitive dyscontrol; PSWQ: Penn State Worry Questionnaire; Suicidality: BDI-II 9 item (suicide); BDI: Beck Depression Inventory-II; PDSS: Panic Disorder Severity Scale; RSPBC: randomized shortest paths to betweenness centrality.

Figure 3 shows the centrality indices for strength, EI, and RSPBC for each node in the networks of PD patients with and without SA. Strength, EI (one-step and two-step), and RSPBC centrality graphs in the PD+SA network showed that AS4 (FCD) was the most central node, followed by BDI. In contrast, the node with the highest strength, EI in one- and two-steps, and RSPBC in the PD-SA network was the BDI, followed by AS1 (fear of respiratory symptoms). In addition, AS4 (FCD) and BDI exhibited the highest eigenvectors in PD+SA. In PD-SA, the highest eigenvector was the BDI, followed by PSWQ.

The results for the accuracy of all the edge weights are presented in Figure S1. The two estimated networks in patients with PD were proven to be within the margin of error: the CIs of the strongest edge weights did not overlap the CIs of other edge weights. In addition, the stability for strength (CS‐C = 0.52), EI (CS‐C = 0.52), and RSPBC (CS‐C = 0.60) was observed at a high level. The eigenvector (CS‐C = 0.44) showed an appropriate level of stability. The PD-SA network demonstrated an excellent stability level (CS‐C = 0.75 for strength and EI, 0.75 for RSPBC, and 0.59 for eigenvector, respectively) (Figure S2). The bootstrapped edge weight difference test results are shown in Figure S3.

#### 3.5.2. Network Comparison between PD Patients with and without Suicide Attempts

In terms of centrality, the PD+SA group had significantly higher strength (diff = 1.25, *p* FDR = 0.025), EI (diff = 0.66, *p* FDR = 0.022), and eigenvector (diff = 1.36, *p* FDR < 0.001) in AS4 (FCD), RSPBC in the AS4 (FCD) (diff = 1.38, *p* FDR = 0.032), and suicidality (diff = 0.68, *p* FDR = 0.008), as shown in Figure 3. The PD-SA group showed significantly higher strength (diff = 0.96, *p* = 0.041) and eigenvector (diff = 1.06, *p* FDR = 0.007) in the AS3 (fear of cardiovascular symptoms). Furthermore, the PD-SA group showed higher strength (diff = 1.27, *p* FDR = 0.025), EI (diff = 1.13, *p* FDR = 0.005), and eigenvector (diff = 2.09, *p* FDR < 0.001) in the PSWQ.

Conversely, the two edge weight scores were significantly higher for PD-SA than for PD+SA: (1) BDI–PSWQ (diff = 0.28, *p* FDR = 0.028) and (2) AS1 (fear of respiratory symptoms)–AS3 (fear of cardiovascular symptoms) (diff = 0.20, *p* = 0.034).

## 4. Discussion

To the best of our knowledge, this is the first study to suggest that an SA history is a potential predictor of short- and long-term pharmacological treatment of PD after controlling for age, sex, frequency of early trauma, AS level, and symptom severity at baseline. In the network analysis, FCD was the most common symptom in patients with PD+SAs who received pharmacotherapy only.

This study presented that the PD+SA group showed a significantly younger age of onset than that of the PD group without SAs. Furthermore, after controlling for age, the frequency of trauma, level of AS, and symptom severity were significantly higher in PD patients with SAs than in those without SAs. In meta-analyses, younger age of onset and a high level of depressive symptoms were associated with an increased risk of SAs in patients with PD. There were associations between suicidal ideation and clinical variables, including younger age of onset, longer duration of illness, comorbid depressive disorder, and agoraphobia in patients with PD [14]. Furthermore, a high frequency of early trauma [62] and increased levels of AS [19, 63] were significantly correlated with a high level of suicidality in patients with PD. Recently, it was reported that the higher the level of pathological worry, the greater the risk of SAs in patients with affective disorders [12]. Our results are consistent with those of previous studies.

Our findings using multiple linear regression analysis suggested that the presence of SAs was a significant predictor of short- and long-term pharmacological treatment after adjusting for age, sex, level of trait anxiety, frequency of early trauma, and symptom severity at baseline in patients with PD. There are several predictors of unfavorable short- and long-term pharmacological treatment, such as female sex, lower age at onset, longer duration of illness, frequency of early trauma [64], higher panic symptom severity score, higher levels of pathological worry [65], and comorbid depression in patients with PD [66]. However, the presence of SAs has not been reported as a predictor of short- and long-term pharmacological treatment outcomes in patients with PD.

The cause of these findings remains unknown. However, patients in the PD+SA group showed severe symptoms, high levels of AS, and comorbid agoraphobia compared to those without SAs, which might be related to poor pharmacological treatment outcomes. Even after controlling for these variables, SA history was found to be a significant unfavorable predictor of short-term and long-term pharmacological treatment in patients with PD. Biologically, impulsivity with a high level of AS is a crucial risk predictor of SA behavior in patients with affective disorders [67]. Chronic impulsivity, which is related to 5-HT disturbance, might be explained by the vulnerability to suicidality and correlated with a high level of trait anxiety and aggressive behavior in patients with PD [68]. These biological vulnerabilities in the PD+SA group might have reduced the short-term and long-term unfavorable pharmacological treatment response.

In our network analysis, FCD was the strongest central symptom in the PD+SA group. Tests of network comparison showed significant differences in the FCD values as centrality indices such as strength, EI, and RSPBC in the PD+SA group compared to the PD-SA group. Furthermore, in the PD+SA group, the FCD was found to be the most central symptom in the plots of centrality, including the two-step EI, RSPBC, and eigenvector. These results suggest that the significant symptom node (*FCD*) might affect the other two or more two-step connected nodes (*depression and suicidality*). That is, it suggests that the FCD might be able to affect suicidality through depression, panic symptoms, or depression through other nodes. Thus, it seems that the network approach can show their relationship, suggesting that both depression and FCD are associated with suicidality in PD+SA. Therefore, FCD may be the central and important symptom of PD symptomatology in the PD+SA group.

The FCD is significantly correlated with the severity of suicidality in patients with anxiety disorders [24]. Furthermore, a high level of FCD was significantly associated with an increased risk of SAs in patients with anxiety disorders [69]. The higher the FCD in patients with PD, the greater the fear of losing control over mental capacities, which might increase susceptibility to suicidal behavior [70]. This finding is similar to that of a depression study which showed that escalated catastrophic cognitions related to cognitive AS and panic symptoms are associated with SAs in major depression. Although a high level of depressive symptoms can greatly impact SAs, it seems that FCD is more central than depressive symptoms in patients with PD+SA in network analysis.

In addition, pharmacotherapy may improve depressive and anxiety symptoms in patients with PD. However, cognitive AS symptoms do not improve with continuous pharmacotherapy in patients with PD+SA. In addition to pharmacotherapy monotherapy, CBT intervention is needed to reduce cognitive AS symptoms in patients with PD+SA [71, 72]. In other words, it may be more effective to accompany pharmacotherapy with CBT, such as interoceptive exposure, a cognitive reconstruction that can improve dysfunctional beliefs, psychoeducation, or mindfulness, for the treatment of patients with PD+SA [73, 74].

Our study showed that patients with PD + SA undergoing pharmacotherapy, compared with PD-SA, displayed characteristics, such as an earlier onset of panic symptoms, more severe PD-related psychopathology, and poorer short- and long-term pharmacological treatment responses. These findings imply that clinicians should be vigilant to the possibility that the presence of SA in their patients with PD may predict an unfavorable short- and long-term pharmacological treatment outcome. Furthermore, network analyses revealed that cognitive AS could be the most central factor as a potential target symptom in patients with PD+SA. Therefore, our study suggests that the cognitive aspects of AS in patients with PD+SA may be clinically effective as potential targets for intervention in patients with PD who are at risk of or suffer from suicidality.

This study had several limitations. First, there were reports of bias that assessed the history of suicidality among the participants in this study. However, we supplemented this with other clinical assessments such as the BDI-II and SSI. Second, patients with multiple SAs were not analyzed in our study. A follow-up study is needed to compare the pharmacological treatment responses and clinical characteristics according to the frequency of SAs in patients with PD. Third, to evaluate the short- and long-term treatment responses, our study included patients with PD who received only pharmacotherapy and not psychotherapy. Therefore, our findings may differ from those of patients with PD who receive combination therapy or psychotherapy in clinical practice. Fourth, our study included patients whose principal diagnosis was PD, except for depressive disorders with psychotic features, which might have led to a selection bias. This could limit the generalization of the findings of our study in the clinical setting. Causality was not inferred from the network analysis results. Further studies are required to determine the causality of SAs in patients with PD undergoing pharmacotherapy.

## 5. Conclusion

In conclusion, this study showed that SA history might increase the risk of unfavorable short- and long-term pharmacological treatment outcomes in patients with PD after controlling for age, sex, symptom severity, AS level, and frequency of early trauma at baseline. Furthermore, the present study suggests that it is important to clinically assess the history of suicidality, cognitive AS, and symptom severity at baseline when determining the direction of treatment in patients with PD, especially in PD+SA. Additionally, CBT will be helpful for clinicians in reducing the FCD in patients with PD+SA.

## Figures and Tables

**Table 1:** Sociodemographic and clinical characteristics of the patients with PD and HCs.

Variables	PD (*n* = 755)	HCs (*n* = 396)	Statistics	*p* value
	Mean (± SD) or *n* (%)	Mean (± SD) or *n* (%)	*t* or *χ*^2^	
Sex	360 (47.70)	187 (47.20)	0.02	0.882
Men/women	/395 (52.30)	/209 (52.80)		
Age (years)	38.78 (±11.40)	37.60 (±10.87)	1.70	0.086
Marital status				
Living with partner	452 (60.80)	190 (52.80)	6.47	0.012
Living without partner	291 (39.20)	170 (47.20)		
Duration of illness (months)	40.67 (±65.93)	n/a	n/a	n/a
Baseline SSI total score	5.39 (±6.79)	1.79 (±3.10)	6.36	<0.001⁣^∗∗^
Baseline PDSS total score	10.91 (±6.29)	0.15 (±0.68)	31.66	<0.001⁣^∗∗^
8-week PDSS total score	9.84 (±5.06)	n/a	n/a	n/a
6-month PDSS total score	8.68 (±4.84)	n/a	n/a	n/a
1-year PDSS total score	7.61 (±4.45)	n/a	n/a	n/a
Baseline BDI-II total score	16.23 (±10.34)	5.23 (±4.93)	19.12	<0.001⁣^∗∗^
Baseline PSWQ total score	52.85 (±12.56)	38.78 (±9.59)	12.77	<0.001⁣^∗∗^
Baseline ASI-R total score	48.88 (±28.92)	8.66 (±10.78)	24.99	<0.001⁣^∗∗^
Fear of respiratory symptom	18.20 (±11.73)	1.44 (±2.70)	26.30	<0.001⁣^∗∗^
Fear of publicly observable anxiety reaction	10.66 (±7.56)	3.41 (±4.32)	19.56	<0.001⁣^∗∗^
Fear of cardiovascular symptom	13.67 (±9.55)	2.97 (±5.04)	16.59	<0.001⁣^∗∗^
Fear of cognitive dyscontrol	6.39 (±6.48)	0.81 (±1.73)	15.76	<0.001⁣^∗∗^
Baseline ETISR-SF total score	4.76 (±3.92)	3.02 (±2.79)	5.16	<0.001⁣^∗∗^

Note: ⁣^∗∗^*p* < 0.001. Values are reported as count (percent), mean ± SD. Abbreviations: n/a: not available; SD: standard deviation; PD: panic disorder; HCs: healthy controls; SSI: Scale for Suicidal Ideation; PDSS: Panic Disorder Severity Scale; BDI-II: Beck Depression Inventory-II; PSWQ: Penn State Worry Questionnaire; ASI-R: Anxiety Sensitivity Index-Revised; ETISR-SF: the Early Trauma Inventory Self Report-Short Form.

**Table 2:** Sociodemographic and clinical characteristics of the patients with PD with and without the history of suicide attempt.

Variables	PD+SA (*n* = 97)	PD–SA (*n* = 658)	Statistics	
	Mean (± SD) or *n* (%)	Mean (± SD) or *n* (%)	*t* or *χ*^2^	*p* value
Sex	42 (43.30)	318 (48.30)	0.86	0.355
Men/women	/55 (56.70)	/340 (51.70)		
Age (years)	33.84 (±11.42)	39.51 (±11.23)	4.64	<0.001⁣^∗^
Duration of illness (months)	45.61 (±44.33)	40.18 (±48.34)	0.99	0.320
Family history of anxiety disorder (yes)	29 (30.90)	160 (24.90)	1.51	0.219
Use of antidepressants, *n* (%)	72 (84.70)	542 (90.30)	2.54	0.110
Paroxetine	36 (42.40)	354 (59.00)		
Escitalopram	31 (36.50)	175 (29.20)		
Sertraline	3 (3.50)	10 (1.70)		
Venlafaxine	3 (3.50)	8 (1.30)		
Antidepressants equivalent dose of (mg/day)^a^	11.28 (±8.72)	10.08 (±6.78)	-1.226	0.223
Onset age of panic symptoms (years)	26.09 (±11.27)	35.82 (±11.22)	7.53	<0.001⁣^∗^
Agoraphobia^b^	31.00 (±19.97)	19.07 (±16.11)	-6.53	<0.001⁣^∗^
Suicidality				
SSI total score	14.49 (±8.18)	3.83 (±5.09)	-11.96	<0.001⁣^∗^
BDI-II item 9	0.99 (±0.84)	0.21 (±0.45)	-13.76	<0.001⁣^∗^

Note: values are reported as count (percent), mean ± SD. ⁣^∗^*p* < 0.001. ^a^The approximate equivalent oral dose to 10 mg escitalopram is given. ^b^The agoraphobia means the APPQ-agoraphobia subscale scores. Abbreviations: SD: standard deviation; PD: panic disorder; SA: history of a suicide attempt; SSI: Scale for Suicidal Ideation; BDI-II: Beck Depression Inventory-II.

**Table 3:** Results of ANCOVA analyses for controlling age between patients with PD with and without a past history of suicide attempt.

Variables	PD+SA (*n* = 97)	PD–SA (*n* = 658)	Statistics		
	(mean ± SD)	(mean ± SD)	*F*	df	*p* value
Baseline PDSS total score	14.03 (±6.51)	10.45 (±6.13)	20.06	1	<0.001⁣^∗∗^
8-week PDSS total score	13.38 (±5.53)	9.29 (±4.76)	28.15	1	<0.001⁣^∗∗^
6-month PDSS total score	12.38 (±4.99)	8.15 (±4.59)	25.48	1	<0.001⁣^∗∗^
1-year PDSS total score	10.72 (±4.89)	7.23 (±4.24)	14.91	1	<0.001⁣^∗∗^
Baseline BDI-II total score	25.57 (±11.76)	14.82 (±9.34)	89.20	1	<0.001⁣^∗∗^
Baseline PSWQ total score	59.11 (±12.34)	52.00 (±12.36)	9.67	1	<0.001⁣^∗∗^
Baseline ASI-R total score	67.03 (±30.86)	46.15 (±27.63)	34.55	1	<0.001⁣^∗∗^
Baseline ETISR-SF total score	7.83 (±4.49)	4.33 (±3.64)	27.01	1	<0.001⁣^∗∗^

Note: values are reported as mean ± SD. ⁣^∗∗^*p* < 0.001. Abbreviations: ANCOVA: analysis for covariance; SD: standard deviation; PD: panic disorder; SA: history of a suicide attempt; PDSS: Panic Disorder Severity Scale; BDI-II: Beck Depression Inventory-II; PSWQ: Penn State Worry Questionnaire; ASI-R: Anxiety Sensitivity Inventory-Revised; ETISR-SF: the Early Trauma Inventory Self Report-Short Form.

**Table 4:** Results of multiple regression analysis to predict treatment response for patients with panic disorder.

Factor	Treatment response at 8 weeks *R*^2^ = 0.19^∗^ (*n* = 450)		Treatment response at 6 months *R*^2^ = 0.20^∗^ (*n* = 379)		Treatment response at 1 year *R*^2^ = 0.22^∗^ (*n* = 329)	
	*β*	*p* value	*β*	*p* value	*β*	*p* value
Sex	0.10	0.150	0.14	0.088	0.08	0.392
Age	-0.06	0.436	0.04	0.622	0.01	0.943
Baseline PDSS total score	0.46	<0.001⁣^∗∗^	0.48	<0.001⁣^∗∗^	0.42	<0.001⁣^∗∗^
Baseline BDI-II total score	0.05	0.649	0.09	0.472	0.17	0.185
Baseline PSWQ total score	-0.07	0.466	-0.11	0.286	-0.18	0.094
Baseline ASI-R total score	-0.14	0.185	-0.19	0.102	-0.19	0.110
Baseline ETISR-SF total score	0.07	0.337	0.04	0.600	0.11	0.236
A history of the suicide attempt	-0.19	0.011⁣^∗^	-0.20	0.015⁣^∗^	-0.28	0.002⁣^∗^

Note: model *p* values < 0.001. ⁣^∗^*p* < 0.05. ⁣^∗∗^*p* < 0.001. Abbreviations: PD: panic disorder; SA: suicide attempt; PDSS: Panic Disorder Severity Scale; BDI-II: Beck Depression Inventory-II; PSWQ: Penn State Worry Questionnaire; ASI-R: Anxiety Sensitivity Inventory-Revised; ETISR-SF: the Early Trauma Inventory Self Report-Short Form.

**Table 5:** Demographics and response frequencies for clinical assessment of participants in the network analyses.

Variable	PD+SA group (*n* = 97)	PD–SA group (*n* = 485)
	Frequency (%)	Frequency (%)
Sex		
Male	42 (43.3%)	206 (42.5%)
Female	55 (56.7%)	279 (57.5%)
Age (years)		
Mean ± SD	33.84 ± 11.42	35.74 ± 10.41
PDSS		
0-10 (mild)	31 (32.0%)	259 (53.4%)
11-15 (moderate)	26 (26.8%)	133 (27.4%)
16-28 (severe)	40 (41.2%)	93 (19.2%)
BDI-II		
0-13 (minimal)	14 (14.4%)	251 (51.8%)
14-19 (mild)	20 (20.6%)	109 (22.5%)
20-28 (moderate)	26 (26.9%)	89 (18.3%)
29-63 (severe)	37 (38.1%)	36 (7.4%)
Item 9 (suicide item)		
0 (no suicidal ideation)	28 (28.9%)	392 (80.8%)
1 (mild)	48 (49.5%)	93 (19.2%)
2 (moderate)	15 (15.4%)	0 (0.0%)
3 (severe)	6 (6.2%)	0 (0.0%)
PSWQ		
16-39 (low)	3 (3.1%)	55 (11.3%)
40-59 (moderate)	37 (38.1%)	292 (60.2%)
60-80 (high)	57 (58.8%)	138 (28.5%)
ASI-R		
Fear of respiratory symptom		
*T*‐score < 65	82 (84.5%)	458 (94.4%)
*T*‐score ≥ 65	15 (15.5%)	27 (5.6%)
Fear of observable anxiety reaction		
*T*‐score < 65	75 (77.3%)	463 (95.4%)
*T*‐score ≥ 65	22 (22.7%)	22 (4.5%)
Fear of cardiovascular symptom		
*T*‐score < 65	80 (82.5%)	436 (89.9%)
*T*‐score ≥ 65	17 (17.5%)	49 (10.1%)
Fear of cognitive dyscontrol		
*T*‐score < 65	72 (74.2%)	447 (92.2%)
*T*‐score ≥ 65	25 (25.8%)	38 (7.8%)

Note: values are reported as mean ± SD. Abbreviations: SD: standard deviation; PD: panic disorder; SA: suicide attempt; BDI-II: Beck Depression Inventory-II; PDSS: Panic Disorder Severity Scale; PSWQ: Penn State Worry Questionnaire; ASI-R: Anxiety Sensitivity Inventory-Revised.

## Data Availability

The datasets generated and analyzed during the current study are not publicly available due to legal or ethical restrictions that protect patients' privacy and consent but available from the corresponding authors on reasonable request.

## Supplementary Materials

Supplementary Materials Supplementary material for this article is available online. Supplementary Figure 1: accuracy of edge weight parameters and their bootstrapped confidence intervals. Supplementary Figure 2: estimation of the edge weight bootstrapped difference test result in patients with panic disorder (a) with and (b) without the history of a suicide attempt. Supplementary Figure 3: stability of the centrality indices by case dropping subset bootstrap.
