# Supplementary material for: Poor Treatment Response in Panic Disorder Patients with Suicide Attempts and Their Symptom Network Characteristics
Source: Depress Anxiety. 2023 May 4;2023:5194900. doi: 10.1155/2023/5194900 (PMC11921835; doi:10.1155/2023/5194900)
Supplement: Supplementary Materials — Supplementary material for this article is available online. Supplementary Figure 1: accuracy of edge weight parameters and their bootstrapped confidence intervals. Supplementary Figure 2: estimation of the edge weight bootstrapped difference test result in patients with panic disorder (a) with and (b) without the history of a suicide attempt. Supplementary Figure 3: stability of the centrality indices by case dropping subset bootstrap. [file 5194900.f1.docx]

**Supplementary Materials**

**Supplementary Figures**

**
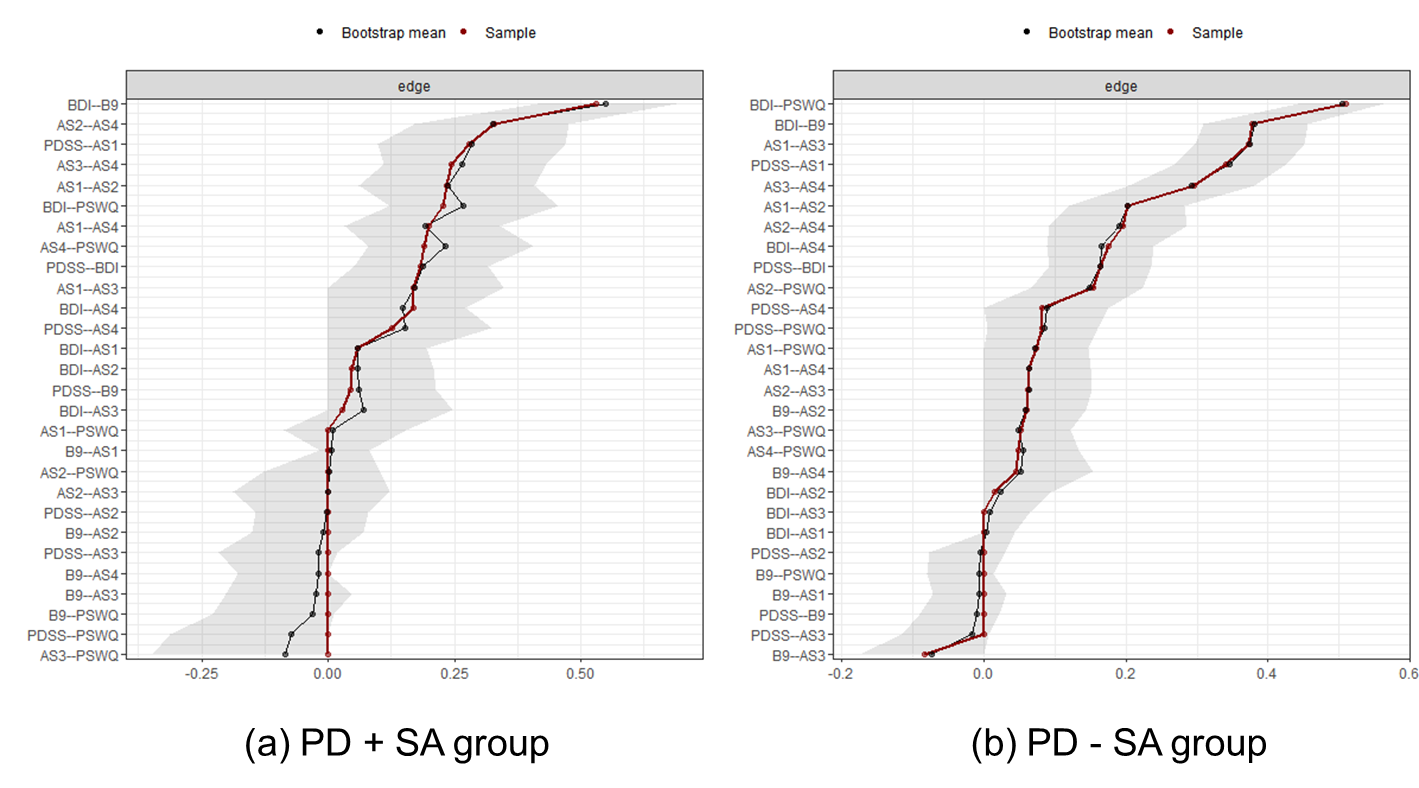
**

**Figure S1. Accuracy of edge weight parameters and their bootstrapped confidence intervals.** The gray area indicates the 95% confidence intervals for each edge. The red line represents the edges, and the black line indicates the bootstrapped mean. The y-axis represents all observed edges of the network. Abbreviations: PD, panic disorder; SA, suicide attempt; AS1, fear of respiratory symptom; AS2, fear of publicly observable anxiety reaction; AS3, Fear of cardiovascular symptom; AS4, fear of cognitive dyscontrol; PSWQ, Penn State Worry Questionnaire; Suicidality, BDI-II 9 item (suicide); BDI, Beck Depression Inventory-II; PDSS, Panic Disorder Severity Scale.


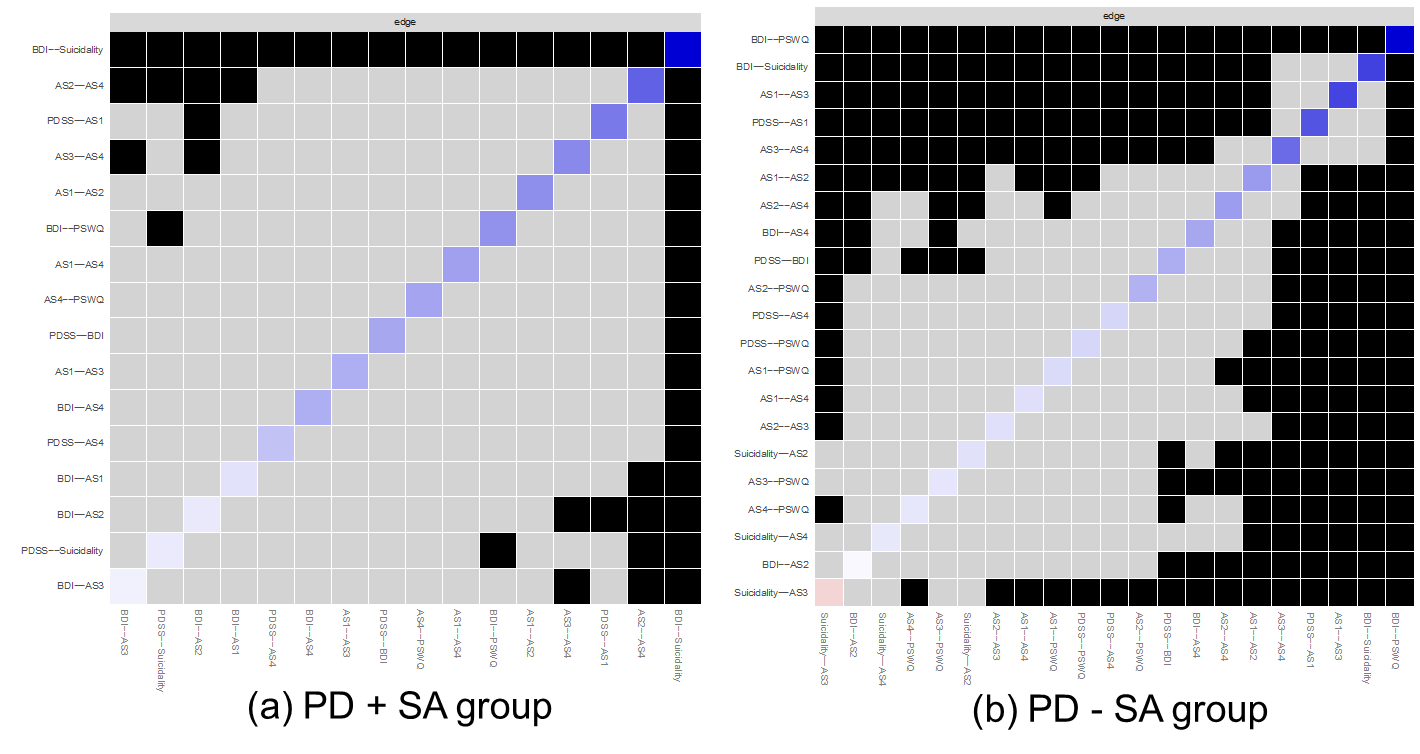


**Figure S2. Estimation of the edge weight bootstrapped difference test result in patients with panic disorder (a) with and (b) without the history of a suicide attempt.** The black boxes show the edges that differ significantly from each other. The gray boxes show the edges that do not significantly differ from each other. The number of black boxes is higher in PD-SA than in PD+SA, indicating that the stability in the network is relatively high. Abbreviations: PD, panic disorder; SA, suicide attempt; AS1, fear of respiratory symptom; AS2, fear of publicly observable anxiety reaction; AS3, Fear of cardiovascular symptom; AS4, fear of cognitive dyscontrol; PSWQ, Penn State Worry Questionnaire; Suicidality, BDI-II 9 item (suicide); BDI, Beck Depression Inventory-II; PDSS, Panic Disorder Severity Scale.

**
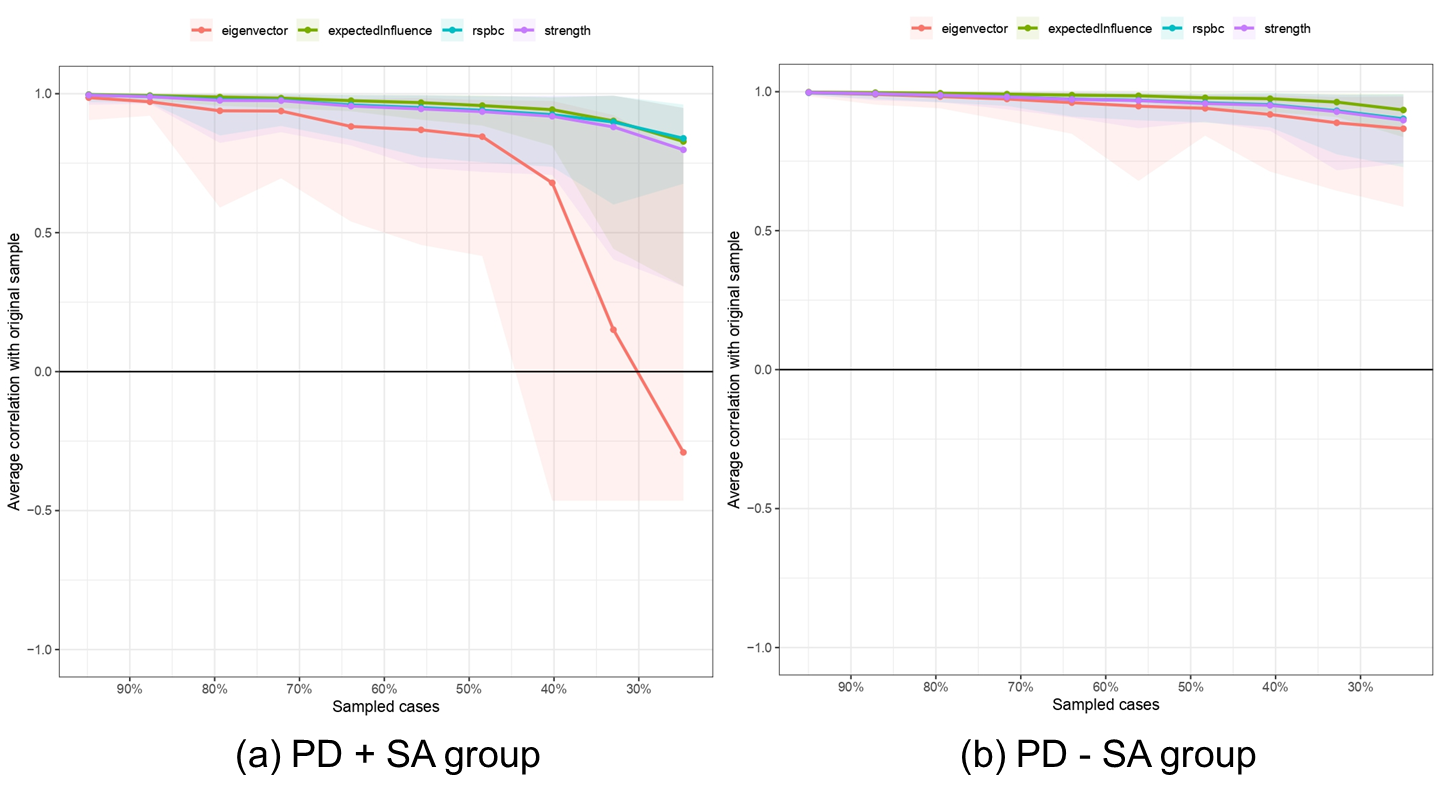
**

**Figure S3. Stability of the centrality indices by case dropping subset bootstrap.** The x-axis represents the percentage of cases of the original sample used at each step. The y-axis represents the average of associations between the centrality indices from the original network and that from the re-estimated networks after dropping increasing percentages of cases. Each line indicates the correlations expected influence, strength, randomized shortest paths betweenness centrality, and eigenvector, while areas indicate a 95% confidence interval. Abbreviations: PD, panic disorder; SA, suicide attempt.
